# Planning the City Logistics Terminal Location by Applying the Green *p*-Median Model and Type-2 Neurofuzzy Network

**DOI:** 10.1155/2016/6972818

**Published:** 2016-04-19

**Authors:** Dragan Pamučar, Ljubislav Vasin, Predrag Atanasković, Milica Miličić

**Affiliations:** ^1^Department of Logistics, University of Defence in Belgrade, Pavla Jurisica Sturma 33, 11000 Belgrade, Serbia; ^2^Faculty of Technical Science, University of Novi Sad, Dositeja Obradovića 6, 21 000 Novi Sad, Serbia

## Abstract

The paper herein presents green *p*-median problem (GMP) which uses the adaptive type-2 neural network for the processing of environmental and sociological parameters including costs of logistics operators and demonstrates the influence of these parameters on planning the location for the city logistics terminal (CLT) within the discrete network. CLT shows direct effects on increment of traffic volume especially in urban areas, which further results in negative environmental effects such as air pollution and noise as well as increased number of urban populations suffering from bronchitis, asthma, and similar respiratory infections. By applying the green *p*-median model (GMM), negative effects on environment and health in urban areas caused by delivery vehicles may be reduced to minimum. This model creates real possibilities for making the proper investment decisions so as profitable investments may be realized in the field of transport infrastructure. The paper herein also includes testing of GMM in real conditions on four CLT locations in Belgrade City zone.

## 1. Introduction

Since logistics operators are stressed by increased demands, distribution centers and terminals are usually situated in the vicinity and/or within the urban areas. By introducing the “just-in-time” concept, numerous production systems have reduced their needs for warehousing facilities that required significant costs for regular and major maintenance. The result was that segment of the inventory was introduced into the transport system thus contributing to heavy traffic congestions and pollution in urban areas including negative effects on both environment and society. Empirical researches in Great Britain that included a sample of 87 companies have confirmed that 39% of the companies have experienced reduction in both number and capacity of their warehouse facilities, while 1/3 of the companies recorded an increased volume of delivery transport [1].

Research performed on cooperation and consolidation systems that use logistics terminal showed that numerous companies reduced their costs in the range of 5% to 20%. By applying these systems in urban areas, vehicle per kilometer rate is reduced for 60%. Decrement of road freight movements by 30% to 60% resulted in reduction of heavy vehicle fleet. In addition, delivery volume was increased for 15%, while noise and emission of harmful gases were reduced accordingly [2].

CLT are established on traffic-favorable locations, on the perimeter of the city or in the city core areas where they coordinate entry and exit flows as well as flow of goods between the point of origin and the point of consumption. Big cities are provided with special logistics terminals that correspond to the city logistics domain, so that the logistics centers become major component of the system for goods supply and elimination of waste. Number, size, and location of logistic terminals depend on size and characteristics of the city.

Consequences of CLT location decision, whether they are positive or not, are long-term in character since they considerably affect the efficiency and effectiveness of the analyzed distribution center. Numerous models for determining locations within the network have been developed over recent years. This paper analyzes discrete location problems based on *p*-median problem. The basic assumption of all *p*-median models is to determine location for one or several terminals within the network with the aim to minimize the average distance between the terminals and end-users.

The *p*-median problem was introduced by Hakimi [3]. Some earlier papers on the subject are Babich [4], Love and Morris [5], for rectilinear distances, Cooper [6], Chen [7], Drezner [8] for Euclidean distances, Sherali and Tuncbilek [9] for squared Euclidean distances, and Bongartz et al. [10] for general distances. Love [11] solved the problem on a line using dynamic programming.

A survey of metaheuristic approaches for solving the *p*-median problem is presented in Mladenović et al. [12]. Reese [13] summarized the literature on solution methods for the incapacitated and capacitated *p*-median problem on a network and presented annotated bibliography of different solution methods.

Brimberg and Drezner [14] proposed several heuristics for solving the *p*-median problem. One of the approaches is IALT which is a modification of Cooper's alternate algorithm [6, 15]. Brimberg et al. [16] developed the reformulation local search (RLS) which is a new local search that iterates between the continuous problem and a discrete approximation. Drezner and Marcoulides [17] also proposed a constructive algorithm START, a decomposition approach, and a local search IMP that provide better results than the Cooper like approaches [6, 14, 15].

In the field of metaheuristic solution methods, Houck et al. [18] and Correa et al. [19] proposed the genetic algorithms. Brimberg et al. [20] adopted the genetic algorithm developed by Houck et al. [18], except that they considered sparsity of the median locations and avoided duplication of good locations. Salhi and Gamal [21] proposed a genetic algorithm in which they used new strategies for genetic operators and improved the final solutions. Salcedo-Sanz et al. [22] proposed several hybrid genetic algorithms to solve the capacitated *p-*median problem. Ant colony optimization algorithm [23], grouping harmony search algorithm [24, 25], clustering search metaheuristic [26], bionomic algorithm [27], and variable neighborhood [28] are some of the notable studies, which have been conducted in the recent years.

By analyzing the available literature, it can be noticed that the majority of *p-*median models deals with location planning problem taking into account economic, technical, and technological indicators. However, the said models are not concerned with reducing of adverse effects on environment and people's health as a result of logistics activities. On the other hand, there are certain models that consider environmental parameters when defining location for logistics facilities. The majority of these models has been developed during the past few years. Li et al. [29] propose a biobjective model to optimize distribution center locations by minimizing transportation cost and transportation/production carbon emissions. Mallidis et al. [30] present a multiobjective model to evaluate the effects of different scenarios such as distribution network locations, outsourcing transportation, and warehousing operations on environment. The multiobjective optimization model is proposed by Wang et al. [31] as the supply chain network design considers cost of transportation, handling, and green technology acquisition. They measure the CO_2_ emissions produced by production and distribution facilities. Diabat and Simchi-Levi [32] suggest a mixed integer programing model for supply chain design with limitation of produced CO_2_ in facilities. They prove that the supply chain cost would increase if they put more limitation on produced CO_2_. Harris et al. [33] consider transportation cost and CO_2_ emissions for optimizing European automobile industry. Chaabane et al. [34] propose a mixed integer linear programming based model for sustainable supply chain design with the consideration of life cycle assessment principles and material balance constraints at each node of supply chain.

This paper analyzes GMP which uses the adaptive type-2 neural network for processing of environmental and sociological parameters and costs of logistics operators showing the influence the said parameters have on CLT location planning within the network. The problem refers to defining the CLT location within the discrete network to minimize harmful effects of logistics delivery vehicles to environmental and human health in urban areas. This approach becomes more important considering the fact that in the future it may be expected that transport companies, particularly in countries with developed industries, will have increased number of EFVs in their fleets while number of ENF vehicles will be accordingly reduced.

CLT leads to increment of traffic volume in urban areas. The increased traffic volume directly affects the environment contributing to air pollution and noise while indirectly it is associated with adverse health effects, including bronchitis, asthma, and similar respiratory infections. Therefore, local city authorities make remarkable efforts to include as many low-emission vehicles as possible and to displace logistics activities outside the city core areas. However, there has been a noticeable lack of reliable methodology to support such implementation. In order to optimize the “green” capacity, a system that enables support in decision-making process for the city logistics planning problem has been developed. The purpose of the paper is to propose a model for CLT location taking into account environmental parameters on the studied location and in urban areas in general.

The problem is presented as one of nonlinear optimization with fuzzy values of the input parameters and is solved by using type-2 neurofuzzy model (T2NFM). The advantage of the model lies in the fact that it considers a number of factors that affect the input variables. In the first phase of GMM application, once the possible CLT locations have been defined together with the possible users within the city network, links between users and locations within the network will be identified. PL (performance of link) shall be defined for every link within the network by applying T2NFM. The input parameters for defining PLs within the network are logistics operating costs, environmental parameters (emission of sulfur and nitrogen oxides, carbon monoxide, particulate matters, and noise), and social parameters (number of people suffering from bronchitis, asthma, and similar respiration infections). T2NFM input parameters will be broken down into components that further describe the environmental status, social parameters, and logistics operating costs. The advantage of this model is reflected in the fact that it takes into account a significant number of factors that affect the input variables. For example, when considering the input variable (environmental parameters), the parameters taken into account are those which describe oxides of sulfur emissions, nitrogen oxide emissions, carbon monoxide emissions, and particulate emissions. By summing up the weighted values of the said parameters, the input variable environmental parameters may be described. A similar approach is applied for breaking down the remaining T2NFM input variables (sociological parameters and logistics operating costs). After running the input parameters through the adaptive neural network, PLs are obtained at the output for every link in the network.

After obtaining the PLs values of the network, routes for both EFV and ENF vehicles of the logistics operators, from the CLT to the end destination, may be allocated. Routes shall be defined by applying the modified Dijkstra's algorithm. Once the EFV and ENF routes have been defined, PLs for every CLT location will be summarized and the most optimum location will be chosen. A detailed description of the developed model is stated in the text below. In addition to description of the phases of the model, the architecture of the T2NFM is presented, the input parameters are defined, and the process of training the network is shown. The final section of the paper shows how the model was tested in real conditions on four CLT locations in Belgrade.

## 2. Green *p*-Median Model for CLT Location Planning within the Discrete Networks

The paper analyzes problem referring to selection of CLT location at the city perimeter so as logistics operating costs and environmental and sociological parameters may be reduced to minimum. For the purpose of CLT location planning, modification of the standard *p*-median problem is carried out. Optimal routes to nodes (users) within the network will be allocated for any potential CLT location. As a result, two routes for every CLT location will be allocated: one route for EFVs and the other one for EUF vehicles. In the next phase, performance of all links within the optimum routes, from CLT to nodes (users), within the network will be summarized. The optimum task is to choose the CLT location characterized by minimum total sum of PLs for EUF vehicles and maximum total sum of PLs for EFVs. The result is ranking of CLT locations and selection of the optimum route for transport means, from CLT to nodes (users).

**Figure 1:**
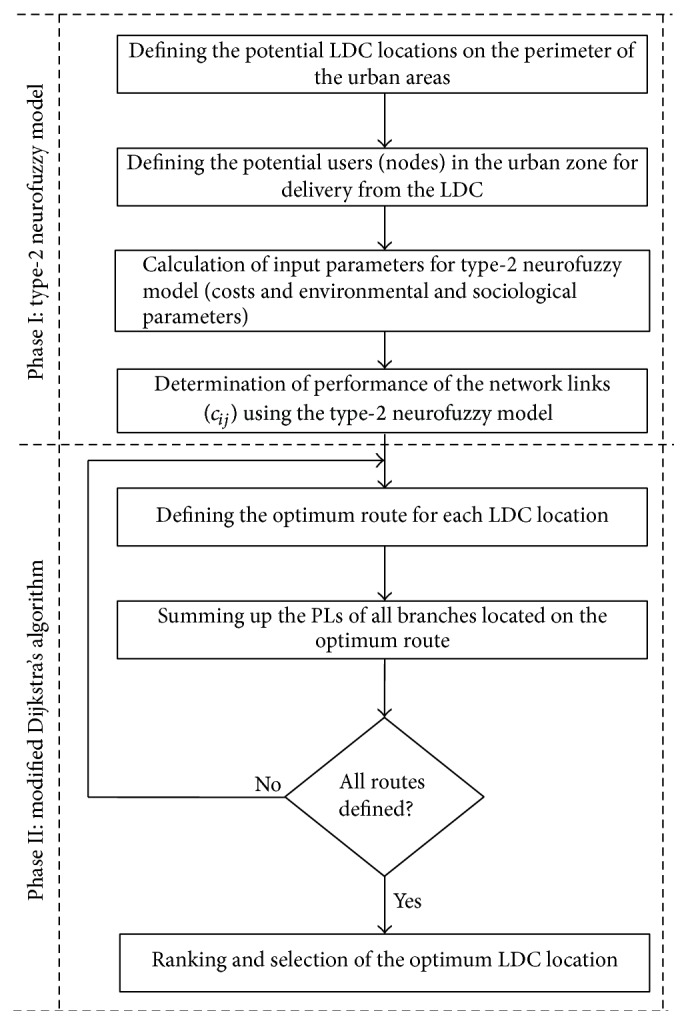
CLT location planning model.

The paper presents solution of *p*-median problem by applying type-2 neurofuzzy model and modified Dikstra's algorithm (Figure 1). Planning of CLT locations at the perimeter of the urban areas is considered to be an optimization problem in a function of logistics operating costs and environmental and sociological parameters. Solution of the problem is proposed through application of T2NFM and modified Dijkstra's algorithm.

CLT location planning shall be done in two phases. In phase one, after defining the potential CLT locations and the users of logistic services, the input parameters of the T2NFM shall be calculated. After application of the T2NFM, the performance of the network links was obtained by passing the input parameters (*x*_1_: costs of the logistics distributors, *x*_2_: environmental parameters, and *x*_3_: sociological parameters) through the adaptive neurofuzzy network.

In phase two, we assign the PL values to network branches and define the optimal routes of EFV and EUV transport means from each CLT location to the users. Delivery vehicle routes shall be determined by application of modified Dijkstra's algorithm. EFV and EUV routing performed by application of modified Dijkstra's algorithm consists of the following steps.

**Step 1 .**

In step one, the initial network node shall be determined. In the model presented in this paper, the initial network nodes (CLT locations) are predefined. The process starts from the node *L*. Since the PL from the node *L* to the node *L* is zero, the initial node is assigned a label PL_*L*_ = 0. The predecessor node of the node *L* will be denoted by the symbol + so that *q*_*L*_ = + (where *q*_*i*_ is the node before the node *i* on the shortest path between the node *L* and the node *i*).

**Step 2 .**

Since the paths from the node *L* to all other nodes are still unknown, we temporarily label PL_*Li*_ = *∞* for *i* ≠ *L*. Since the predecessor nodes *i* of the nodes *i* ≠ *L* on the shortest paths are also unknown, we put *q*_*i*_ = − for all *i* ≠ *L*. The only node that is currently in a closed state is the node *L*. Therefore, we can write that *c* = *L*.

**Step 3 .**

In order to transform some of temporary labels into the permanent ones, we should examine all branches (*c*, *i*) coming from the last node in a closed state (node *c*). If the node *i* is in a closed state, we shall proceed with examination of the next node. If the node *j* is in an open state, we obtain its label based on the equation

(1) $$\begin{array}{c} {\text{PL}}_{cj}=\min\left\{\;{\text{PL}}_{j},{\text{PL}}_{ac}+\text{PL}\left(\;c,j\;\right)\;\right\}. \end{array}$$

**Step 4 .**

In order to determine a next node that will pass from an open to a closed state, we shall compare the values of all nodes in an open state.

For EUV vehicles, we choose a node with the lowest PL value. Let it be the node *j*. The node *j* passes from an open to a closed state considering that there is no PL value between *a* and *j* lower than PL_*aj*_ (2). Link performance through any other node would be higher:

(2) $$\begin{array}{c} {\text{PL}}_{aj}=\max\left\{\;{\text{PL}}_{aj}\;\right\}. \end{array}$$

For EFV, we choose a node with the highest PL value. Let it be the node *j*. The node *j* passes from an open to a closed state considering that there is no PL value from *a* to *j* higher than PL_*aj*_ (3). Link performance through any other node would be lower:

(3) $$\begin{array}{c} {\text{PL}}_{aj}=\min\left\{\;{\text{PL}}_{aj}\;\right\}. \end{array}$$

**Step 5 .**

Since the node *j* is the next node which passes from an open to a closed state, we determine the predecessor node of the node *j* on the shortest path from the node *a* to the node *j*. We examine the link performance of all branches (*i*, *j*) going from the nodes in a closed state to the node *j* until we confirm that (4) is satisfied:

(4) $$\begin{array}{c} {\text{PL}}_{ai}={\text{PL}}_{aj}-\text{PL}\left(\;i,j\;\right). \end{array}$$

Let this equation be satisfied for the node *t*. It means that the node *t* is the predecessor of the node *j* on the shortest path between the node *a* and the node *j*. Therefore, we can write that *q*_*i*_ = *t*.

**Step 6 .**

If all nodes in the network are in a closed state, we have completed the process of finding the optimum EFV and EUV routes. If there are other nodes in an open state, we shall return to Step 3.

**Step 7 .**

After calculation of routes from all potential CLT locations to the users, PLs on the optimum EFV and EUV routes shall be summed up for each CLT location separately:

(5) QEFVi=∑j=1nPLj,i=1,2,…,m,

(6) QENFi=∑j=1nPLj,i=1,2,…,m,

where *Q*_EFV*i*_ is the sum of PLs in the EFV route, *Q*_ENF*i*_ is the sum of PLs in the EUV route, *n* is the total number of links on the optimum routes, and *m* is the total number of CLT locations to be chosen.

**Step 8 .**

Criterion functions (*L*_*i*_) assigned to each location, which serve to rank locations, shall be obtained as a difference between the sum of PLs on the optimum EFV (*Q*_EFV*i*_) and EUV (*Q*_ENF*i*_) routes. Location with the highest difference shall be chosen:

(7) $$\begin{array}{c} L_{i}=Q_{\text{EFV}i}-Q_{\text{ENF}i}. \end{array}$$

The text below describes a process for defining the input parameters and the T2NFM architecture.

## 3. Architecture of Adaptive Type-2 Neurofuzzy Network

An integral part of the adaptive type-2 neurofuzzy network is type-2 fuzzy logic system (T2FLS) of reasoning. The initial T2FLS is mapped into a five-layer adaptive neural network with a restricted connectivity structure.

Based on the analysis of the presented literature and recommendations given by Jovanović et al. [35] and Ćirović et al. [36], parameters which have influence on the routing of logistics vehicles and CLT location planning in city areas have been identified. After passing the defined parameters through the T2NFM, we obtain the performance of the network links. Three input variables of the T2NFM (*x*_1_: costs of logistics distributors, *x*_2_: environmental parameters, and *x*_3_: sociological parameters) were defined.

The mathematical formulation of the T2NFM input variables is made in the following way. Parameter for the costs of the logistics distributors (*x*_1_) is determined on the basis of operating costs of the vehicle (*c*_1_), route length (*l*), vehicle driving time on the link (*t*), and staff costs (*c*_2_), namely, *x*_1_ = *c*_1_ *l* + *c*_2_ *t*  [35].

Environmental parameters (*x*_2_) were used to analyze the air quality and noise. Emitted quantities of sulfur oxide (*E*_1_), nitrogen oxide (*E*_2_), carbon monoxide (*E*_3_), and particulate matters (*E*_4_) were considered as representative chemical compounds illustrating the air condition. In addition to those chemical compounds, a noise (*E*_5_) was considered due to its harmful impact on the environment and human health. Each environmental parameter is assigned a weighting factor *ω*_*i*_ (*i* = 1,2,…, 5). After summing up the weighted environmental parameters (∑_*i*=1_^5^ *ω*_*i*_ · *E*_*i*_), we obtain the environmental parameter (*x*_2_) that passes through the adaptive T2NFM.

Values of the above-mentioned chemical compounds are obtained from the measuring stations placed in the cities or from the reports prepared by the environmental protection agencies. Values from the SEA report [37] were used in this paper.

For determination of the noise parameters, there are numerous mathematical models that illustrate a traffic noise with different accuracy and each one is different with regard to the factors taken into consideration. All models that can be found in the literature are based on establishing a functional relation between the noise emission parameters and the parameters related to traffic and roads/streets [36]. Some of the most widely used models were defined by Burgess [38], Josse [39], and Fagotti and Poggi [40]. In this research, we used a model for determination of equivalent noise level generated by the road traffic, which has been developed by Prascevic et al. [41].

Sociological parameters (*x*_3_) were used to analyze a number of people sick with bronchitis (*S*_1_), asthma (*S*_2_), and respiratory infections (*S*_3_) represented by a number of sick people per 1000 population. Each sociological parameter is assigned a weighting factor *ω*_*i*_. After summing up the weighted sociological parameters (∑_*i*=1_^3^ *ω*_*i*_ · *S*_*i*_), we obtain a sociological parameter (*x*_3_) that passes through the T2NFM.

Values of these parameters depend on the economic situation and economic policy of each country where the proposed algorithm could be applied. Based on the data obtained from the Serbian Environmental Protection Agency, we defined the limit values *E*_1_  [0,125] *μ*g/m^3^, *E*_2_  [0,85] *μ*g/m^3^,  *E*_3_  [0,10] *μ*g/m^3^, and *E*_4_  [2,40] *μ*g/m^3^ [36].

*Adaptive Type-2 Fuzzy Logic Model Training*. Since the output of the network is numeric, then this can be compared with the expected output from a teacher (i.e., supervised learning) and backpropagation used to feed the error back to adjust the parameters in the nodes.

The procedure in the adaptive type-2 fuzzy logic system is made up of forward and backward passes while using a combined steepest gradient descent and least square error method for the learning process required in determining the parameters' values for the adaptive nodes [42, 43].

**Figure 2:**
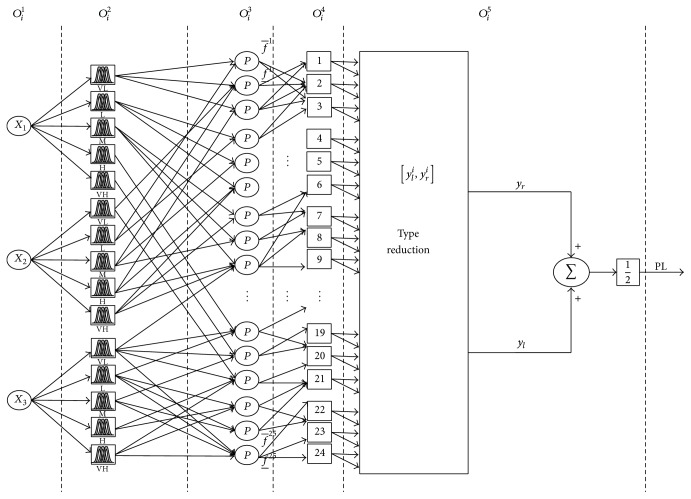
The structure of type-2 neurofuzzy model.

Adaptive type-2 fuzzy logic system is designed to establish and compute a function from input space to output space. In this paper, the network (Figure 2) that has a fixed structure is configured based on the operation of the fuzzy system (Takagi-Sugeno model). The input layer (Layer 1) consists of 3 units representing costs of the logistics distributors (*x*_1_), environmental parameters (*x*_2_), and sociological parameters (*x*_3_). This is the input layer for the network. The net input and the net output for the *i*th node of this layer are indicated as

(8) $$\begin{array}{c} O_{i}^{\left(1\right)}=w_{i}^{\left(1\right)}x_{i}^{\left(1\right)}, \end{array}$$

where the weights of *w*_*i*_^(1)^  (*i* = 1,2,…, *n*) are set to be unity and *x*_*i*_^(1)^ is the input to the *i*th node in this first layer.

**Figure 3:**
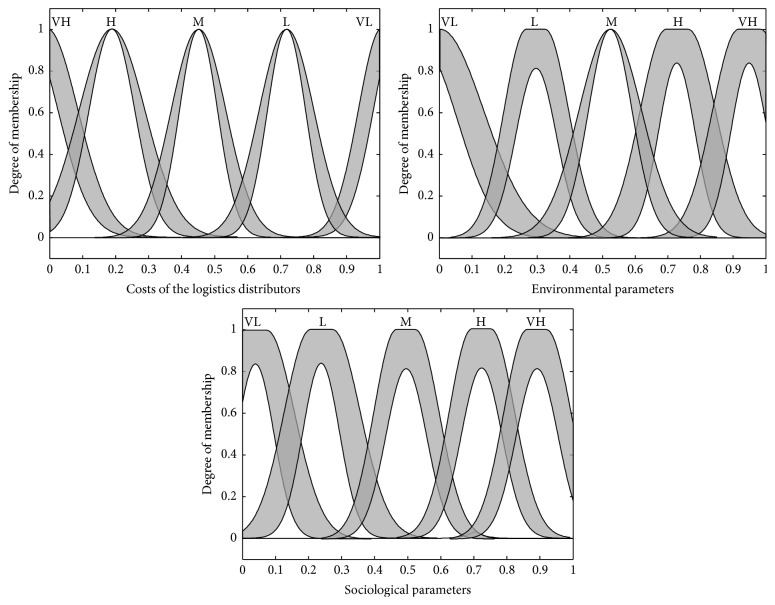
Membership functions for the T2NFM.

Every node in 2nd layer is an adaptive node. This layer handles the type-2 membership functions. The nodes here consist of linguistic membership grades to be matched with the input variables used in the training process. All the units in the 2nd layer (*x*_1_, *x*_2_, and *x*_3_) are connected with the 5 units. The 2nd layer consists of 3 + 5 units representing the number of verbal descriptions quantified by type-2 fuzzy sets (“very low,” “low,” “medium,” “high,” and “very high”) for each input variable (Figure 3). Every unit in the 2nd layer is an adaptive unit with an output being the membership value of the premise part.

Two types of MF are adopted here. In this paper, we have used the type-2 Gaussian MFs with uncertain mean and type-2 Gaussian MFs with uncertain standard deviation to be the membership function for the antecedent and consequent variable. Based on these two MFs, the two kinds of output from this layer are presented in Cases 1 and 2 as follows.

**Case 1 .**

This is the case involving the antecedent and consequent membership functions that have been defined to be Gaussian MF, having uncertain mean (*m*). The output for this case is of the following form:

(9) Oij2exp⁡−12Oij1−mijσij2=O¯ij2as  mij=m¯ijO_ij2as  mij=mij.

**Case 2 .**

This is the case involving the input membership functions that have been defined to be Gaussian MF having uncertain standard deviation (*σ*). The output from this case is as follows:

(10) Oij2exp⁡−12Oij1−mijσij2=O¯ij2as  σij=σ¯ijO_ij2as  σij=σij.

The type-2 MFs can be represented as interval bound by the upper MF and the lower MF, which is denoted as ${\bar{\mu }}_{{\tilde{F}}_{i}}$ and ${\underline{\mu }}_{{\tilde{F}}_{i}}$, respectively; therefore, the output from the 2nd layer is represented as an interval [${\underline{O}}_{ij}^{\left(2\right)},{\bar{O}}_{ij}^{\left(2\right)}$], where ${\underline{O}}_{ij}^{\left(2\right)}$ represents the output from the lower MF and ${\bar{O}}_{ij}^{\left(2\right)}$ is the output from the upper MF.

The third layer is the layer where the operations involving fuzzy rules are carried out. The antecedent matching takes place in this layer following the type-2 fuzzy rules. The operation in this layer is implemented as product (meet) operation. Thus, the output from this layer for the *j*th rule node is as follows:

(11) $$\begin{array}{c} O_{ij}^{\left(3\right)}=\overset{n}{\underset{i=1}{\prod }}\left(\;w_{ij}^{\left(3\right)}O_{ij}^{\left(2\right)}\;\right)=\left\{\begin{array}{cc} {\bar{O}}_{ij}^{\left(3\right)}=\overset{n}{\underset{i=1}{\prod }}\left(w_{ij}^{\left(3\right)}{\bar{O}}_{ij}^{\left(2\right)}\right) & \\ {\underline{O}}_{ij}^{\left(3\right)}=\overset{n}{\underset{i=1}{\prod }}\left(w_{ij}^{\left(3\right)}{\underline{O}}_{ij}^{\left(2\right)}\right), & \end{array}\right. \end{array}$$

where the weights *w*_*ij*_^(3)^ are set to be unity. Therefore, similar to layer 2, the output *O*_*ij*_^(3)^ from this layer is represented as an interval [${\underline{O}}_{ij}^{\left(3\right)},{\bar{O}}_{ij}^{\left(3\right)}$].

The node in 4th layer is responsible for the generation of the rules' output by carrying out consequent matching. The “join” operation (Union) regarding the grades of membership also takes place in this layer:

(12) $$\begin{array}{c} O_{ij}^{\left(4\right)}=\overset{n}{\underset{i=1}{\prod }}\left(\;w_{ij}^{\left(4\right)}O_{ij}^{\left(3\right)}\;\right)=\left\{\begin{array}{cc} {\bar{O}}_{ij}^{\left(4\right)}=\overset{n}{\underset{i=1}{\prod }}\left(w_{ij}^{\left(4\right)}{\bar{O}}_{ij}^{\left(3\right)}\right) & \\ {\underline{O}}_{ij}^{\left(4\right)}=\overset{n}{\underset{i=1}{\prod }}\left(w_{ij}^{\left(4\right)}{\underline{O}}_{ij}^{\left(3\right)}\right). & \end{array}\right. \end{array}$$

The meet (intersection) operation is used to connect the antecedents in the fuzzy rules; the extended sup-star composition is used to combine the output fuzzy sets and firing strength of the input fuzzy sets, while the join (Union) operation is used to combine multiple rules [42]. The membership grades of type-2 FLS are usually represented by the upper and lower membership grades of the footprint of uncertainty (FOU) [44]. To illustrate the extended sup-star composition using the type-2 fuzzy rules *R* ^*l*^ *:  IF  X*_1_ *is  F*_1_^*l*^ *and  X*_2_ *is  F*_2_^*l*^ *and…and  X*_*n*_ *is  F*_*p*_^*l*^ *THEN  y is  G* ^*l*^, as a hypothetical case, let $\mu_{F_{i}^{l}}=[{\underline{\mu }}_{F_{i}^{l}},{\bar{\mu }}_{F_{i}^{l}}]$ and let $\mu_{G^{l}}=[{\underline{\mu }}_{G^{l}},{\bar{\mu }}_{G^{l}}]$ for each of the samples (*x*, *y*). The type-2 FLS' firing strength given as *μ*_*F*^*l*^_(*x*) = ⋂_*i*=1_^*n*^ *μ*_*F*_*i*_^*l*^_(*x*) is an interval; that is, $\mu_{F^{l}}(X)=[{\underline{f}}^{i}(X),{\bar{f}}^{i}(X)]$. Given that the interval type-2 FLS made use of meet operation under product *t*-norm, therefore the firing strength, as presented below in (14), will be an interval type-1 fuzzy set [44]:

(13) $$\begin{array}{c} f^{i}\left(\;X\;\right)=\left[\;{\underline{f}}^{i}\left(\;X\;\right),{\bar{f}}^{i}\left(\;X\;\right)\;\right]=\left[\;{\underline{f}}^{i},{\bar{f}}^{i}\;\right], \end{array}$$

where

(14) f_iX=μ_F1jx1×⋯×μ_Fnjxn,

(15) f¯iX=μ¯F1jx1×⋯×μ¯Fnjxn,

where × represents the *t*-norm product operation.

**Figure 4:**
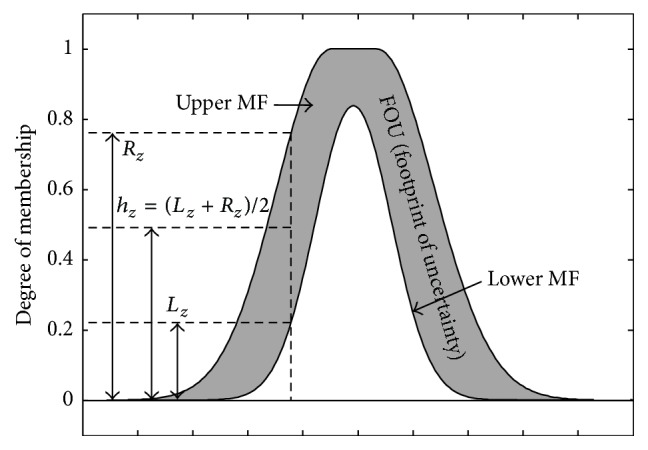
Computation of the parameters *y* ^*t*^,  *y*_*r*_^*t*^, and *y*_*l*_^*t*^.

In the 5th layer, the type reduction and final defuzzification take place. The only node here is a fixed node. The results from the inference engine are type-2 fuzzy sets. There is then the need to reduce the type-2 fuzzy sets to type-1 fuzzy sets in order to give room for defuzzification, so that the final crisp outputs can be generated. Center-of-sets (COS) type-reducer algorithm developed by Mendel [42] has been used in this study because it provides reasonable computational complexity compared to others, such as the expensive centroid type reducer, though other types can still be investigated when the need arises as the research progresses. COS type reducer uses two steps in reducing the type-2 fuzzy sets as follows: (i) calculating the centroids of type-2 fuzzy rule consequences and (ii) calculating the reduced fuzzy sets. Suppose that the output of an interval type-2 FLS is represented by type-2 fuzzy sets *G* ^*t*^ (where *t* = 1,…, *T*,  *T* is the number of output fuzzy sets). In this first stage, the centroids of all the *T* output fuzzy sets are calculated and they will be used in calculating the reduced sets in the next stage. The centroid of the *i*th output fuzzy set *y* ^*t*^ is a type-1 interval set which can be expressed in the following equation [42]:

(16) $$\begin{array}{c} y^{t}=\left[\;y_{l}^{t},y_{r}^{t}\;\right]=\int_{\theta_{1}\in Jy_{1}}\int_{\theta_{z}\in Jy_{z}}\frac{1}{\sum_{z=1}^{Z}y_{z}\theta_{z}/\sum_{z=1}^{Z}y_{z}}, \end{array}$$

where *y*_*r*_^*t*^ and *y*_*l*_^*t*^ stand for the upper bound and lower bound point of *y* ^*t*^ and *θ*_*z*_ is set to *h*_*z*_ that has been defined in Figure 4 and is representing the number of discretized points for each output fuzzy set.

To calculate the type-reduced set, it is sufficient to compute its upper and lower bounds of the reduced sets *y*_*l*_ and *y*_*r*_, which can be expressed as follows:

(17) yl=∑i=1Mfliyli∑i=1Mfli,yr=∑i=1Mfriyri∑i=1Mfri,

where *f*_*l*_^*i*^ and *y*_*l*_^*i*^ are the firing strength and the centroid of the output fuzzy set of *i*th rule associated with *y*_*l*_, respectively. Similarly, *f*_*r*_^*i*^ and *y*_*r*_^*i*^ are the firing strength and the centroid of the output fuzzy set of *i*th rule associated with *y*_*r*_, respectively.

The final output of type-2 FLS is thus set to the average of *y*_*l*_ and *y*_*r*_:

(18) $$\begin{array}{c} y\left(\;x\;\right)=\frac{y_{l}+y_{r}}{2}, \end{array}$$

where *y*(*x*) is the final crisp output.

T2NFM has been trained with 3110 numerical data sets (training pairs). During the training, data from the training set are passed through the network periodically. The T2NFM training was carried out in 890 epochs. When the training process started, the T2NFM output error was 2.584. After 890 epochs, the error was reduced to the acceptable 0.112.

**Figure 5:**
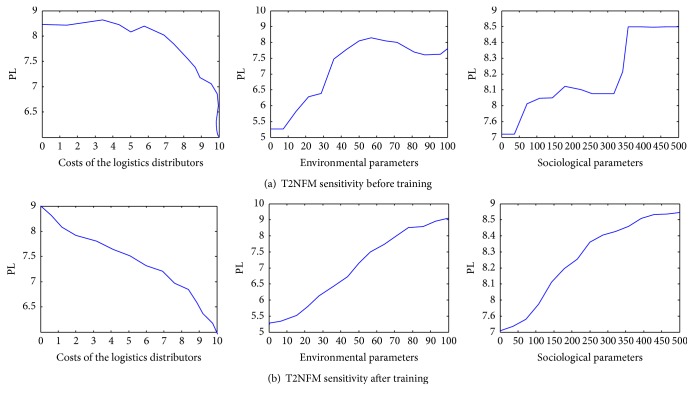
T2NFM sensitivity before and after training.

After training, it was noted that the system is sensitive and the output is gradual. The inert and hypersensitive parts of the system were removed, as it was the case with the type-2 fuzzy model (Figure 5(a)).  Figure 5(b) shows the system sensitivity by input parameters after training, namely, a scenario that describes the system reaction for some input values.

Five-layer adaptive network was tested on the example of 178 parameters describing the network nodes in the urban areas of Belgrade. Values of the criteria describing the observed node were periodically passed through the T2NFM and the network node PLs were obtained.

## 4. The Model Testing

**Figure 6:**
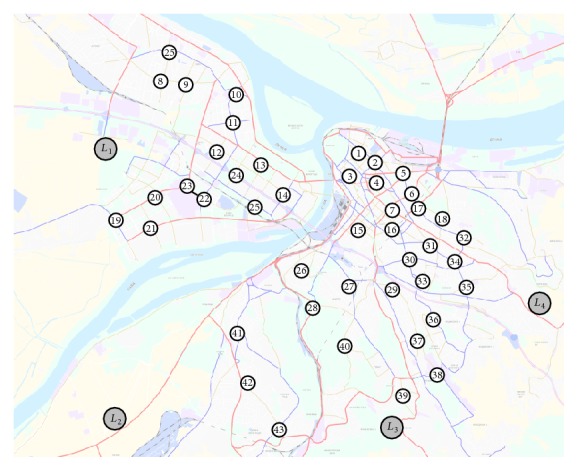
CLT locations and network architecture.

The GMM was tested in the urban areas of Belgrade. The significance of the goods transport in Belgrade is presented by the research results [45] showing that two-thirds of the total goods flows have the source or destination in the central urban areas. The Central Business District initiates one-third of all freight flows [45]. Urban freight transport has continual growth and there are expectations that the trend will continue in the future. A reason for the growing share of the freight transport in Belgrade is the trends in production and distribution based on the low stock and the deliveries that are precisely defined in time (JIT, just-in-time strategy) as well as the trend of growth of electronic trade and the delivery to the home address (B2C, Business-to-Customer). Four traffic-suitable locations for the CLT development were chosen on the city core periphery (Figure 6). Figure 6 shows the network of main roads connecting the CLT locations and 43 service users.

Traffic congestion, which is very present in the so-called peak traffic periods, is extremely harmful to the environment and causes many negative consequences such as additional fuel consumption, air pollution, supply problems, reduced trade, and effects of transport vehicles. Moreover, most of the harmful gases (about 1/3) are produced by the vehicles, namely, 65% of carbon monoxide, 45% of hydrocarbon, and 49% of nitrogen oxide [46].

In order to inform the public about the air quality in Belgrade, the Environmental Protection Agency monitors the air quality and noise in real time. For this purpose, automatic measuring stations were placed in Belgrade to monitor the air quality. The SEA report [37] warns that parameters of the environment in Belgrade are significantly worse as compared to the previous years. In addition, the report of the Belgrade Institute of Public Health [47] states that the number of people sick with asthma, bronchitis, and respiratory infections is growing and one of the main reasons for such situation is deteriorated air quality.

Having in mind the data presented in the reports of the Belgrade Institute of Public Health [47] and the SEA [37], the Belgrade City authorities have decided to subsidize the purchase of EFV in order to reduce the environmental pollution in the city. The goal for the next four years is that logistics distributors shall have minimum 50% EFV in their fleets. However, in addition to purchasing the EFV, the logistics distributors will face the problem of how to allocate the EFV vehicles. In addition to CLT location planning, the GMM presented in this paper proposes the routes of EFV and EUV vehicles serving to supply the CLT users.

As stated in Section 2, the CLT location planning shall be made in two phases. In phase 1, after defining the potential CLT locations and the users of logistic services (Figure 6), the input variables of the T2NFM shall be calculated. The input variables of the T2NFM were obtained based on the limit values for the period 2010–2013 [37, 47]. Parameters of the variable *x*_2_ (environmental parameters) were obtained based on the data collected from 35 automatic measuring stations for monitoring the air quality and noise [37]. Parameters of the variable *x*_3_ (sociological parameters) were obtained based on the report of the Belgrade Institute of Public Health [47] while parameters of the variable *x*_1_ (costs of the logistics distributors) were obtained based on the analysis of the costs of the logistics distributors and Euclidean length of links of the network where the model was tested.

Characteristics of the links, namely, parameters of the variables *x*_1_, *x*_2_, and *x*_3_ on the tested network, are shown in Table 1 (the Appendix). Based on the values shown in Table 1, the input variables of the T2NFM were obtained. After passing the variables (*x*_1_: costs of the logistics distributors, *x*_2_: environmental parameters, and *x*_3_: sociological parameters) through the adaptive neural network, we obtain the performance of the network links. At T2NFM output 178 PLs shown in Table 1 were obtained.

In the second phase, the PL values are assigned to the network branches and the optimum routes of EFV and EUV transport means from each CLT location to the users are defined. Using the modified Dijkstra's algorithm, we have obtained two routes for each CLT location. EFV and EUV routes were defined for each CLT location. Using expressions (5) and (6) for each CLT location individually, we sum up the PLs located on the optimum routes. The sums of PLs on the CLT locations in the tested network are shown in Table 2.

After summing up the PLs, we choose a location that has minimum influence on the sociological parameters and environmental pollution, that is, the EFV route with the maximum sum of PLs. In addition, a location shall be chosen based on the costs of the logistics distributors; namely, the EUV route with the minimum sum of PLs will be chosen. A final value of the criterion function associated with the locations shall be obtained using expression (15). Based on the values of criterion functions, we rank locations and choose the best one. The values of criterion functions and rank of locations are shown in Table 2.

**Figure 7:**
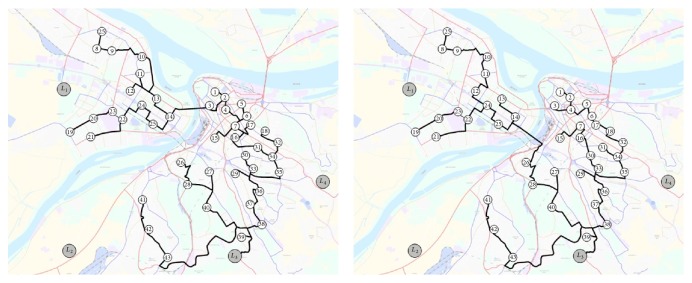
EFV and EUV routes on the location GLT_3_.

After analysis of the obtained results (Table 2), we can conclude that location GLT_3_ has minimum influence on the sociological parameters and the parameters of environmental pollution in the urban area. In addition, the CLT constructed on the location GLT_3_ will provide maximum cost savings for the logistics distributors. EFV and EUV routes on the location GLT_3_ are shown in Figure 7.

When defining the routes, it was assumed that the number of EFVs is limited and that a maximum of ten vehicles can be assigned to one of the routes. Under these assumptions the steps of proposed algorithm were applied and vehicles were assigned to the given routes.

## 5. Discussion and Conclusions

According to the world trends, this paper contains the GMM developed for CLT location planning, whose logistic capacities are used to serve the urban areas. The model was tested in the real Belgrade City network. After using the real data obtained from the automatic measuring stations placed in Belgrade, the parameters for calculation of the input variables of the T2NFM were obtained and model was tested in the real Belgrade City road network. This model remains open for modifications and further adaptation to the user's requirements. This model is adaptive and it is possible to use some other models instead of the proposed method for obtaining the specific input data and that is particularly significant when the input noise parameters and the costs of the logistics operators are being considered. Therefore, instead of the proposed model for determination of noise parameters used in this paper [41], it is possible to use some other model judged as a quality one by the user, which better reflects the situation in the field. Moreover, the developed model structure allows for the possibility of there being the incomplete data. One of the advantages of this model is that it takes into account the uncertainties which arise when predicting the costs of the logistic operators, environmental parameters, and sociological parameters in the city road network. In other words, if specific data on input parameters are not available, the model approximates the values and gives the valid results. It is very important when the input environmental parameters are considered according to harmful gases emission criterion, where the users will often be in situation to have incomplete data due to numerous parameters, which cannot be measured easily. The application of the adaptive neural network makes it possible to continuously incorporate new theoretical and empirical knowledge gained through application in the practice of this model and the similar ones. In addition, the proposed model enables the CLT location planning to maximize the positive environmental impacts, which is reflected in the reduction of harmful gas emissions and in improvement of air quality in the areas with the highest concentration of the population.

This model extends the theoretical framework of knowledge in the area of green logistics. The existing problem is considered using new methodology thus creating a basis for further theoretical and practical upgrading. In addition, the presented model emphasizes the new criteria (environmental parameters, sociological parameters, and costs of the logistics operators), which have not been considered in the former models but are relevant to this issue. By introducing and describing the new criteria in the model, the need for their consideration in the further analysis of this and similar problems is pointed out.

The GMM takes into consideration a fact that the logistics distributors have limited EFV fleet. Therefore, EFV and EUV routes are separately considered in the model. Based on the constructed routes, we choose location with the lowest negative impacts on the environment and health of the population in urban residential areas, which will minimize the costs of the logistics distributors. Likewise, the algorithm supports any number of available EFVs and EUVs, which is consistent with the network size and the transport demand.

This model has been developed to minimize the harmful consequences of the logistics flows in the urban areas on the environment and sociological parameters. Precisely, this makes it compliant with the world trends since traffic in general, but particularly goods transport, has very complex environmental impact resulting in the series of negative effects manifested through air pollution, water pollution, noise, energy consumption, reduced safety, vibrations, and others. On the other hand, biofuels such as biogas, biomethane, and natural gas are increasingly being used and are replacing the traditional fossil fuels. The practical value of this algorithm lies in the fact that the collected experience of numerous experts is incorporated into the model, thus avoiding a situation in which the CLT location planning and vehicle routing are limited to the knowledge of individuals who find themselves in a position to solve these problems alone.

Directions of the future research could be oriented to find the optimum distribution of the locations in order to minimize the effects of logistic processes on the sports and recreation zones, water areas, relaxation and residential zones, green urban areas, and so forth. Heuristic and metaheuristic methods would be adequate for solving the mentioned problem. Such problem could be considered by the genetic algorithm or some other suitable method. In addition, the future research could be oriented to study the influence of stations supplying EFVs and EUVs with driving energy on the CLT location planning. The future research and improvements of this model should take into consideration the problem related to location for filling batteries on EFVs and their optimum location in relation to the CLT location.

## Figures and Tables

**Table 1:** Characteristics of the links in the tested network.

Number	Link	*E*_1_	*E*_3_	*E*_2_	*E*_4.1_	*E*_4.2_	*E*_5_	*S*_2_	*S*_1_	*S*_3_	*l*_*i*_	*t*	*X*_1_	*X*_2_	*X*_3_	PL
1	*R*_*L*1-25_	38.10	4.00	43.80	5.10	2.00	35.00	58.00	62.00	32.00	6.80	11.66	4.86	26.96	52.20	5.38
2	*R*_*L*1-8_	33.00	7.40	62.60	12.00	6.70	32.00	47.00	58.00	65.00	5.70	9.77	4.07	31.46	56.11	5.55
3	*R*_*L*1-9_	18.00	2.40	48.10	19.10	5.30	29.10	70.00	23.00	56.00	6.20	10.63	4.43	24.61	48.69	5.18
4	*R*_1-2_	83.50	7.50	43.20	31.80	15.30	78.00	332.00	362.00	366.00	0.80	1.37	0.57	52.14	352.72	8.67
5	*R*_1-3_	98.50	7.20	66.00	21.60	19.60	80.00	149.00	268.00	185.00	1.00	1.71	0.71	59.33	203.16	8.29
6	*R*_1-4_	62.30	4.60	82.80	26.80	11.30	87.90	258.00	174.00	320.00	1.40	2.40	1.00	57.41	244.44	8.49
7	*R*_1-5_	82.40	5.40	42.30	37.00	3.70	46.60	155.00	71.00	70.00	2.10	3.60	1.50	42.29	100.02	6.12
8	*R*_1-7_	121.80	2.60	79.90	28.90	17.30	33.00	93.00	433.00	154.00	3.20	5.49	2.29	53.86	235.93	8.56
9	*R*_2-3_	115.50	9.80	53.90	34.00	6.90	49.10	418.00	375.00	336.00	0.90	1.54	0.64	52.37	379.28	8.83
10	*R*_2-4_	95.10	8.10	74.40	33.80	19.10	87.30	164.00	449.00	338.00	0.60	1.03	0.43	64.09	318.17	9.20
11	*R*_2-5_	69.80	6.90	12.10	29.50	14.00	59.20	444.00	325.00	198.00	1.10	1.89	0.79	37.67	330.96	7.00
12	*R*_2-7_	98.60	3.00	57.30	35.00	8.10	76.80	201.00	372.00	450.00	1.85	3.17	1.32	56.02	333.92	8.95
13	*R*_3-4_	39.60	5.90	31.20	11.80	13.60	82.10	215.00	432.00	114.00	0.80	1.37	0.57	39.63	267.12	6.70
14	*R*_3-5_	96.40	8.20	72.80	5.60	5.40	52.00	382.00	429.00	237.00	1.30	2.23	0.93	49.37	358.65	8.58
15	*R*_3-7_	122.70	4.40	74.40	10.50	13.60	42.00	250.00	188.00	423.00	1.60	2.74	1.14	52.73	275.57	8.35
16	*R*_3-15_	72.90	5.70	27.90	36.00	3.80	45.70	152.00	229.00	219.00	2.35	4.03	1.68	37.30	199.19	6.36
17	*R*_3-16_	31.40	2.20	67.50	17.30	17.20	98.10	273.00	406.00	382.00	2.42	4.15	1.73	50.30	352.86	8.86
18	*R*_4-5_	66.90	8.00	13.20	26.40	16.40	43.60	45.00	433.00	134.00	0.85	1.46	0.61	33.47	213.59	5.72
19	*R*_4-6_	120.80	8.60	81.50	10.50	6.30	83.50	368.00	387.00	443.00	0.95	1.63	0.68	64.67	395.86	9.59
20	*R*_4-7_	33.40	4.10	25.80	22.40	17.00	56.70	34.00	405.00	445.00	0.82	1.41	0.59	32.19	286.23	6.08
21	*R*_4-15_	119.00	6.50	29.40	11.10	6.00	47.30	338.00	87.00	399.00	1.45	2.49	1.04	43.54	262.28	7.25
22	*R*_4-16_	122.70	3.90	56.40	24.10	14.00	55.60	129.00	300.00	57.00	2.15	3.69	1.54	54.19	172.24	7.98
23	*R*_4-26_	121.80	5.90	40.50	37.00	15.80	93.80	178.00	112.00	315.00	5.80	9.94	4.14	62.98	191.88	9.33
24	*R*_4-27_	57.00	7.30	77.00	8.20	3.50	38.80	123.00	404.00	119.00	6.90	11.83	4.93	39.62	225.72	7.65
25	*R*_5-6_	124.00	5.50	60.10	37.70	5.30	95.50	396.00	445.00	112.00	0.75	1.29	0.54	66.57	334.74	9.43
26	*R*_5-7_	80.00	2.60	63.30	13.40	14.20	94.60	406.00	119.00	442.00	1.10	1.89	0.79	56.44	310.01	8.76
27	*R*_5-15_	72.00	9.00	42.10	34.50	6.20	59.70	396.00	174.00	245.00	2.90	4.97	2.07	44.60	271.70	7.96
28	*R*_5-16_	33.80	7.40	70.90	15.50	5.30	80.10	184.00	219.00	342.00	3.25	5.57	2.32	46.13	241.04	8.01
29	*R*_5-26_	21.10	5.10	78.90	19.10	7.10	49.10	37.00	117.00	390.00	6.87	11.78	4.91	37.66	165.34	6.94
30	*R*_5-27_	51.30	5.40	67.10	31.50	12.20	51.20	55.00	235.00	121.00	7.25	12.43	5.18	43.51	140.09	7.50
31	*R*_6-7_	44.20	5.00	5.30	11.50	16.30	50.10	197.00	159.00	361.00	0.75	1.29	0.54	26.79	228.74	5.30
32	*R*_6-17_	49.70	6.30	44.90	15.50	18.30	34.90	228.00	45.00	211.00	0.45	0.77	0.32	33.22	155.62	5.26
33	*R*_7-15_	63.50	9.00	12.60	30.40	9.80	71.40	33.00	154.00	252.00	1.10	1.89	0.79	39.81	139.16	5.94
34	*R*_7-16_	78.10	9.20	72.30	30.50	14.70	38.50	35.00	89.00	182.00	0.90	1.54	0.64	47.15	96.12	6.44
35	*R*_7-17_	101.40	4.70	54.40	15.00	15.40	53.50	163.00	181.00	127.00	1.20	2.06	0.86	48.56	159.43	7.08
36	*R*_9-10_	49.80	4.60	76.30	13.90	11.40	72.20	237.00	191.00	306.00	1.95	3.34	1.39	48.04	239.27	7.80
37	*R*_9-11_	71.00	7.50	56.40	10.90	10.60	35.20	404.00	317.00	159.00	2.35	4.03	1.68	38.21	303.28	7.24
38	*R*_10-11_	56.80	6.90	16.50	36.70	15.60	85.20	294.00	98.00	129.00	0.45	0.77	0.32	44.17	175.41	6.58
39	*R*_10-13_	64.60	7.10	9.20	11.20	4.30	83.70	85.00	327.00	421.00	1.40	2.40	1.00	38.88	268.48	6.74
40	*R*_8-9_	24.50	7.10	59.70	22.00	15.90	59.80	51.00	437.00	254.00	2.50	4.29	1.79	38.97	250.66	7.00
41	*R*_10-24_	55.90	3.30	42.10	21.40	6.60	41.70	428.00	395.00	345.00	3.20	5.49	2.29	34.13	392.44	7.58
42	*R*_10-14_	98.60	5.50	34.10	11.00	6.60	63.10	69.00	395.00	403.00	3.95	6.77	2.82	44.83	283.07	8.32
43	*R*_10-25_	123.90	7.60	11.40	36.30	16.00	51.00	38.00	261.00	329.00	4.15	7.11	2.96	46.42	202.14	7.93
44	*R*_10-23_	38.40	4.20	73.60	22.80	12.10	95.00	413.00	117.00	242.00	2.85	4.89	2.04	52.54	255.74	8.55
45	*R*_11-12_	79.20	5.80	18.30	21.50	3.40	94.60	195.00	213.00	110.00	0.86	1.47	0.61	47.18	178.02	7.01
46	*R*_11-13_	109.30	3.50	68.50	8.90	16.90	62.80	376.00	290.00	235.00	0.78	1.34	0.56	54.51	304.69	8.50
47	*R*_11-24_	22.40	2.60	30.00	34.50	8.40	37.70	168.00	236.00	228.00	1.10	1.89	0.79	26.27	209.84	5.20
48	*R*_11-14_	64.60	6.40	23.40	35.50	11.60	67.30	149.00	91.00	281.00	2.80	4.80	2.00	41.58	164.66	6.69
49	*R*_11-25_	114.30	4.70	61.40	34.60	10.80	57.60	402.00	159.00	407.00	3.10	5.31	2.21	55.36	313.35	9.12
50	*R*_11-22_	23.00	4.80	42.60	27.60	14.50	75.90	419.00	405.00	422.00	2.15	3.69	1.54	39.36	414.55	7.85
51	*R*_11-23_	50.30	9.10	69.10	27.40	5.40	71.70	446.00	240.00	444.00	2.10	3.60	1.50	48.47	369.28	8.71
52	*R*_12-13_	107.00	7.40	47.20	39.40	4.90	89.60	308.00	372.00	298.00	1.10	1.89	0.79	59.86	328.87	9.08
53	*R*_12-14_	98.70	8.00	33.60	22.10	17.50	73.40	55.00	313.00	337.00	1.40	2.40	1.00	50.80	229.46	7.83
54	*R*_12-24_	103.20	5.40	5.80	9.30	9.30	65.40	208.00	374.00	217.00	0.75	1.29	0.54	40.41	271.93	6.80
55	*R*_12-25_	88.90	6.00	37.70	9.70	11.00	55.40	109.00	447.00	345.00	1.60	2.74	1.14	42.34	300.17	7.48
56	*R*_12-23_	113.00	4.30	74.70	9.60	16.70	85.60	206.00	142.00	401.00	1.30	2.23	0.93	62.66	236.89	8.77
57	*R*_12-22_	27.30	8.80	7.20	7.50	2.40	58.80	159.00	247.00	143.00	1.52	2.61	1.09	24.92	187.06	5.13
58	*R*_13-14_	120.90	2.80	34.50	36.50	14.10	65.90	161.00	60.00	444.00	1.00	1.71	0.71	53.35	202.63	9.17
59	*R*_13-25_	44.30	2.70	67.80	24.80	3.30	45.70	209.00	288.00	195.00	1.36	2.33	0.97	38.36	234.32	6.41
60	*R*_13-3_	115.30	8.70	9.00	10.60	3.70	99.20	206.00	126.00	62.00	2.10	3.60	1.50	52.20	136.24	7.50
61	*R*_13-15_	105.90	3.10	44.10	15.70	4.00	87.80	248.00	219.00	124.00	2.85	4.89	2.04	54.38	202.70	8.36
62	*R*_13-26_	59.80	8.90	38.70	32.50	2.70	32.30	261.00	193.00	383.00	6.89	11.81	4.92	33.81	270.03	7.48
63	*R*_13-27_	94.70	9.90	36.20	23.10	19.80	65.90	185.00	313.00	317.00	7.65	13.11	5.46	49.50	269.18	9.11
64	*R*_13-28_	20.40	9.10	14.80	18.80	3.50	95.20	426.00	364.00	429.00	7.90	13.54	5.64	36.42	403.95	8.70
65	*R*_14-3_	91.00	5.50	50.40	14.00	15.00	75.60	81.00	355.00	269.00	1.90	3.26	1.36	51.58	234.88	8.11
66	*R*_14-15_	55.90	8.70	28.80	38.60	19.10	84.70	64.00	82.00	36.00	2.20	3.77	1.57	47.56	63.04	6.52
67	*R*_14-26_	94.80	7.90	39.60	39.50	5.20	91.40	426.00	73.00	416.00	3.45	5.91	2.46	56.70	292.56	7.83
68	*R*_14-27_	78.10	3.90	52.20	8.60	9.10	77.20	98.00	89.00	291.00	3.90	6.69	2.79	48.25	148.84	7.60
69	*R*_14-28_	99.90	4.80	30.70	13.40	10.00	85.40	109.00	137.00	350.00	4.30	7.37	3.07	50.71	186.74	8.25
70	*R*_14-41_	103.70	6.00	55.10	37.40	13.40	48.90	182.00	408.00	131.00	7.56	12.96	5.40	50.86	251.19	9.10
71	*R*_15-16_	74.50	9.20	15.20	33.20	18.80	48.20	194.00	222.00	324.00	0.80	1.37	0.57	37.93	240.73	6.33
72	*R*_15-26_	72.80	9.20	77.00	34.50	13.90	99.10	50.00	37.00	397.00	1.50	2.57	1.07	63.39	142.42	8.15
73	*R*_15-27_	104.00	5.00	81.40	18.80	5.60	60.50	378.00	406.00	224.00	1.75	3.00	1.25	55.81	345.18	9.01
74	*R*_15-28_	26.20	5.30	80.30	6.90	16.30	81.10	60.00	102.00	438.00	2.30	3.94	1.64	46.85	181.34	7.35
75	*R*_15-29_	63.00	4.70	12.40	40.00	6.10	78.40	63.00	57.00	88.00	2.25	3.86	1.61	41.36	67.99	5.88
76	*R*_15-30_	103.80	6.10	74.90	33.20	2.90	31.10	313.00	65.00	432.00	1.95	3.34	1.39	48.52	254.46	7.92
77	*R*_16-17_	77.40	2.70	51.20	19.50	17.00	92.30	258.00	417.00	49.00	1.00	1.71	0.71	54.02	258.25	8.23
78	*R*_16-27_	43.70	3.50	11.60	37.40	9.50	37.10	192.00	389.00	151.00	1.50	2.57	1.07	26.82	253.44	5.59
79	*R*_16-30_	27.40	7.20	16.90	24.30	12.10	69.50	80.00	152.00	116.00	0.80	1.37	0.57	32.84	116.86	5.05
80	*R*_16-31_	48.90	8.60	51.00	12.50	18.70	80.00	365.00	393.00	210.00	1.00	1.71	0.71	46.26	332.10	8.00
81	*R*_16-*L*4_	50.60	6.20	15.00	14.30	11.30	41.60	384.00	203.00	249.00	5.20	8.91	3.71	27.73	279.22	6.81
82	*R*_17-18_	110.70	9.80	36.10	38.90	14.20	38.10	103.00	169.00	433.00	0.65	1.11	0.46	46.46	219.86	7.19
83	*R*_17-32_	124.10	8.20	28.40	20.30	2.50	41.00	430.00	91.00	401.00	1.10	1.89	0.79	43.73	296.25	7.48
84	*R*_17-*L*4_	74.90	9.40	66.80	13.00	15.10	64.30	246.00	369.00	74.00	5.20	8.91	3.71	49.93	243.15	8.80
85	*R*_18-31_	89.70	7.00	73.60	24.30	8.20	62.00	78.00	353.00	170.00	0.80	1.37	0.57	53.48	205.66	7.81
86	*R*_18-32_	33.70	7.20	51.70	7.50	9.30	94.20	450.00	266.00	357.00	0.60	1.03	0.43	45.19	355.80	8.00
87	*R*_18-*L*4_	78.70	3.50	60.60	15.20	18.00	47.30	380.00	257.00	255.00	4.30	7.37	3.07	44.28	299.47	8.48
88	*R*_20-19_	68.00	8.70	9.20	11.10	7.10	35.30	369.00	424.00	284.00	0.85	1.46	0.61	27.61	365.67	6.11
89	*R*_20-21_	34.30	9.90	27.50	21.00	8.30	55.30	202.00	98.00	82.00	0.75	1.29	0.54	32.31	130.08	5.05
90	*R*_20-25_	110.20	3.00	57.20	28.30	11.90	35.10	334.00	260.00	112.00	2.00	3.43	1.43	46.85	244.41	7.71
91	*R*_20-14_	30.50	4.60	19.30	38.20	14.10	47.80	426.00	336.00	369.00	2.60	4.46	1.86	29.78	376.88	6.85
92	*R*_20-3_	79.50	9.30	40.00	37.60	17.30	52.00	52.00	300.00	220.00	5.40	9.26	3.86	45.42	190.96	7.91
93	*R*_20-26_	42.30	5.20	45.10	5.50	19.60	82.40	77.00	163.00	296.00	6.45	11.06	4.61	42.89	170.15	7.53
94	*R*_21-19_	107.00	8.30	9.80	26.80	14.30	65.60	412.00	226.00	155.00	0.56	0.96	0.40	45.53	271.24	7.42
95	*R*_21-25_	48.20	9.70	82.10	21.70	14.60	75.40	389.00	270.00	295.00	1.85	3.17	1.32	52.36	318.69	8.69
96	*R*_21-14_	27.50	4.10	57.30	28.30	8.80	70.00	168.00	69.00	428.00	2.20	3.77	1.57	40.91	204.07	6.77
97	*R*_21-26_	106.60	5.80	57.90	10.40	14.60	73.10	431.00	319.00	243.00	6.15	10.54	4.39	54.87	336.80	9.58
98	*R*_21-3_	89.90	8.70	19.40	33.10	9.60	32.40	338.00	352.00	125.00	5.00	8.57	3.57	36.13	283.70	7.60
99	*R*_21-15_	77.60	3.80	63.40	33.40	16.10	55.20	112.00	361.00	131.00	5.20	8.91	3.71	48.89	209.34	8.41
100	*R*_21-27_	50.60	5.10	5.40	16.60	17.00	47.10	332.00	94.00	294.00	5.80	9.94	4.14	27.99	233.43	6.51
101	*R*_22-13_	35.80	4.80	80.50	32.70	9.90	87.20	364.00	390.00	102.00	1.40	2.40	1.00	52.60	300.34	8.41
102	*R*_22-14_	26.60	7.40	56.40	34.80	15.80	93.90	256.00	435.00	53.00	2.10	3.60	1.50	49.24	265.46	8.12
103	*R*_22-24_	121.10	9.60	69.10	23.80	4.90	63.00	80.00	278.00	135.00	0.70	1.20	0.50	58.55	168.66	7.92
104	*R*_22-25_	74.70	5.80	72.60	32.80	5.30	89.80	124.00	235.00	126.00	0.80	1.37	0.57	58.26	165.57	7.88
105	*R*_22-21_	102.60	9.30	8.50	7.00	8.20	30.50	251.00	210.00	411.00	0.80	1.37	0.57	32.22	280.59	6.04
106	*R*_22-20_	77.80	3.20	22.80	14.90	16.40	33.20	236.00	275.00	145.00	1.00	1.71	0.71	32.18	225.10	5.71
107	*R*_23-22_	57.10	6.10	35.10	28.00	12.10	53.70	153.00	53.00	438.00	0.35	0.60	0.25	38.15	195.81	5.95
108	*R*_23-20_	123.10	6.90	69.60	21.00	13.90	65.30	91.00	326.00	80.00	0.40	0.69	0.29	59.83	174.97	7.98
109	*R*_24-13_	23.50	5.50	32.90	5.20	16.30	84.00	373.00	37.00	320.00	0.65	1.11	0.46	36.97	233.93	6.18
110	*R*_24-14_	25.70	9.10	50.50	19.60	7.50	58.90	417.00	33.00	180.00	0.80	1.37	0.57	36.07	208.63	5.92
111	*R*_24-25_	71.50	9.90	62.10	7.40	7.30	96.70	316.00	228.00	299.00	0.75	1.29	0.54	55.17	278.58	8.43
112	*R*_24-26_	85.60	6.00	54.40	13.30	5.90	79.30	181.00	387.00	157.00	3.80	6.51	2.71	51.28	250.37	8.66
113	*R*_24-3_	80.40	5.00	57.40	32.60	14.20	75.00	195.00	246.00	223.00	4.30	7.37	3.07	53.19	221.69	8.67
114	*R*_24-15_	101.40	6.30	17.50	24.20	18.10	46.90	368.00	229.00	308.00	5.50	9.43	3.93	41.00	299.77	8.25
115	*R*_24-27_	70.00	6.30	53.00	13.30	6.30	79.90	381.00	167.00	259.00	6.60	11.31	4.71	48.43	267.56	8.94
116	*R*_24-28_	118.20	6.90	66.00	21.00	18.20	81.40	266.00	136.00	170.00	6.90	11.83	4.93	62.93	191.15	9.39
117	*R*_25-8_	97.80	6.30	74.90	24.10	17.70	69.20	98.00	141.00	412.00	4.50	7.71	3.21	58.14	201.72	8.97
118	*R*_25-9_	109.90	7.60	59.00	13.80	5.30	68.40	203.00	49.00	403.00	5.50	9.43	3.93	54.12	201.75	8.86
119	*R*_25-10_	76.40	2.10	59.40	26.50	3.60	95.90	403.00	417.00	168.00	5.40	9.26	3.86	55.54	342.28	9.58
120	*R*_25-14_	43.20	7.20	47.50	34.20	19.60	96.80	383.00	59.00	121.00	0.60	1.03	0.43	51.48	189.61	7.44
121	*R*_25-3_	113.00	9.70	55.30	12.30	17.60	80.50	238.00	239.00	291.00	2.70	4.63	1.93	58.90	253.30	8.99
122	*R*_25-15_	32.40	2.00	45.20	25.70	9.20	52.50	333.00	61.00	318.00	3.30	5.66	2.36	33.95	228.21	6.55
123	*R*_25-3_	122.70	2.20	81.20	21.00	17.60	52.40	218.00	116.00	355.00	2.40	4.11	1.71	58.26	218.64	8.70
124	*R*_25-26_	93.40	5.30	22.10	31.50	18.60	60.80	111.00	213.00	130.00	2.68	4.59	1.91	44.95	153.93	7.02
125	*R*_25-27_	32.70	3.10	51.60	19.40	3.70	71.40	77.00	218.00	179.00	3.15	5.40	2.25	38.94	157.69	6.44
126	*R*_25-28_	66.40	6.00	18.50	8.80	11.90	50.70	176.00	117.00	84.00	3.25	5.57	2.32	33.04	128.25	5.58
127	*R*_26-27_	105.70	5.80	34.90	22.70	5.20	62.50	437.00	295.00	245.00	1.10	1.89	0.79	47.50	330.62	8.16
128	*R*_26-28_	41.50	5.60	9.70	15.50	11.70	45.40	350.00	82.00	419.00	0.65	1.11	0.46	26.07	270.31	5.52
129	*R*_26-41_	58.30	4.80	19.80	16.90	14.90	35.50	348.00	367.00	92.00	1.45	2.49	1.04	29.06	283.44	5.96
130	*R*_26-*L*2_	117.30	4.40	49.80	6.40	2.20	82.80	405.00	38.00	438.00	5.80	9.94	4.14	55.16	278.53	9.38
131	*R*_27-29_	95.30	9.70	60.50	17.30	5.60	36.10	99.00	113.00	346.00	0.65	1.11	0.46	44.36	173.18	6.61
132	*R*_27-28_	65.70	6.70	84.80	27.00	14.90	96.50	137.00	181.00	117.00	0.70	1.20	0.50	61.65	147.61	7.94
133	*R*_27-30_	101.70	6.00	6.90	16.70	11.10	72.80	31.00	288.00	174.00	1.20	2.06	0.86	43.62	166.25	6.54
134	*R*_27-39_	123.00	2.40	50.30	22.50	10.60	47.00	411.00	316.00	169.00	3.85	6.60	2.75	49.75	308.01	8.93
135	*R*_27-40_	47.30	5.90	19.90	8.80	10.90	33.10	298.00	309.00	231.00	1.00	1.71	0.71	25.13	283.40	5.60
136	*R*_28-40_	77.90	9.00	12.40	22.40	16.80	73.60	447.00	98.00	269.00	1.30	2.23	0.93	42.83	267.78	7.17
137	*R*_28-41_	74.20	3.50	53.70	24.70	15.90	77.80	241.00	322.00	358.00	1.60	2.74	1.14	50.91	303.70	8.39
138	*R*_28-*L*2_	70.60	4.10	49.80	29.10	9.10	98.60	103.00	365.00	192.00	6.30	10.80	4.50	54.67	224.89	9.12
139	*R*_29-30_	89.50	7.40	20.30	24.40	12.40	92.40	227.00	388.00	56.00	0.90	1.54	0.64	50.81	238.74	7.80
140	*R*_29-37_	79.40	5.90	42.20	6.00	3.30	99.90	237.00	261.00	140.00	1.40	2.40	1.00	51.63	218.67	7.83
141	*R*_29-40_	35.30	7.40	28.80	11.70	5.60	57.40	71.00	87.00	319.00	1.20	2.06	0.86	31.18	146.26	5.14
142	*R*_30-31_	48.80	6.80	34.70	31.40	12.60	90.90	122.00	191.00	395.00	0.50	0.86	0.36	46.92	223.96	7.27
143	*R*_30-33_	121.10	7.50	51.90	36.00	14.60	45.00	313.00	265.00	295.00	0.35	0.60	0.25	52.61	290.30	8.19
144	*R*_30-34_	54.60	6.70	40.80	10.40	10.60	63.80	279.00	99.00	389.00	0.60	1.03	0.43	39.18	243.36	6.44
145	*R*_31-32_	43.00	4.80	75.20	13.10	3.10	39.20	156.00	245.00	320.00	0.75	1.29	0.54	36.90	234.95	6.19
146	*R*_31-34_	64.80	3.20	52.40	36.90	17.60	56.30	380.00	92.00	237.00	0.50	0.86	0.36	45.11	233.55	7.13
147	*R*_32-34_	53.90	7.40	61.20	35.70	18.80	65.20	367.00	111.00	189.00	0.40	0.69	0.29	48.20	222.53	7.36
148	*R*_32-*L*4_	58.90	2.90	59.20	19.70	12.50	56.80	217.00	156.00	295.00	2.60	4.46	1.86	42.63	216.23	7.21
149	*R*_33-34_	67.60	8.50	52.30	33.30	9.80	38.10	99.00	179.00	80.00	0.75	1.29	0.54	40.51	123.19	5.82
150	*R*_33-35_	79.70	8.30	47.70	24.90	13.30	76.20	186.00	206.00	341.00	0.85	1.46	0.61	50.97	236.80	7.79
151	*R*_33-36_	66.00	5.80	43.90	35.80	7.50	35.20	56.00	104.00	53.00	0.65	1.11	0.46	37.18	73.01	5.27
152	*R*_34-35_	46.90	2.30	65.60	38.30	9.60	49.60	111.00	143.00	49.00	0.50	0.86	0.36	41.81	105.40	5.84
153	*R*_34-*L*4_	68.50	8.40	44.90	5.60	12.80	72.40	188.00	172.00	225.00	2.20	3.77	1.57	44.82	192.58	7.17
154	*R*_35-36_	101.80	8.80	7.20	39.70	16.40	42.00	86.00	124.00	101.00	1.40	2.40	1.00	39.97	104.26	5.73
155	*R*_35-*L*4_	49.90	8.40	77.00	14.00	13.30	76.90	32.00	435.00	275.00	1.65	2.83	1.18	50.53	249.06	8.00
156	*R*_36-35_	48.90	8.40	36.90	10.60	9.70	82.70	36.00	362.00	194.00	1.80	3.09	1.29	42.54	200.81	6.83
157	*R*_36-*L*4_	36.10	7.90	45.60	21.50	3.10	67.40	105.00	119.00	158.00	2.60	4.46	1.86	38.53	125.02	5.96
158	*R*_36-37_	29.20	3.80	30.00	5.80	13.10	61.30	152.00	95.00	59.00	0.70	1.20	0.50	30.78	104.87	4.79
159	*R*_36-29_	24.70	5.70	9.30	22.30	13.70	62.40	68.00	92.00	102.00	1.25	2.14	0.89	28.55	86.40	4.52
160	*R*_37-38_	25.20	5.00	16.50	16.40	11.10	58.00	65.00	119.00	86.00	0.75	1.29	0.54	27.78	90.86	4.44
161	*R*_37-39_	78.50	7.00	82.10	7.40	12.20	54.80	92.00	365.00	447.00	1.40	2.40	1.00	49.71	292.57	8.14
162	*R*_37-40_	16.70	4.20	13.80	11.70	11.10	58.00	121.00	67.00	56.00	2.00	3.43	1.43	24.86	82.82	4.48
163	*R*_37-*L*3_	7.90	6.20	14.60	12.60	17.60	56.40	189.00	111.00	393.00	2.40	4.11	1.71	24.46	217.13	5.52
164	*R*_38-39_	25.00	7.20	15.20	8.20	8.60	36.10	161.00	124.00	85.00	1.35	2.31	0.96	20.85	125.98	4.42
165	*R*_38-40_	25.80	4.40	12.00	10.50	6.80	54.70	55.00	37.00	31.00	2.20	3.77	1.57	24.59	41.62	4.25
166	*R*_38-*L*3_	35.00	3.00	31.90	20.40	9.90	47.60	98.00	115.00	121.00	2.20	3.77	1.57	29.97	110.73	4.99
167	*R*_39-40_	16.60	8.10	18.20	8.90	2.90	36.20	96.00	71.00	98.00	2.45	4.20	1.75	19.53	87.31	4.29
168	*R*_39-*L*3_	6.90	8.60	16.40	16.80	9.30	55.60	99.00	84.00	89.00	0.95	1.63	0.68	24.43	90.65	4.28
169	*R*_40-42_	49.40	4.80	30.10	19.10	2.40	58.70	75.00	95.00	150.00	3.10	5.31	2.21	34.31	103.40	5.52
170	*R*_40-43_	29.50	3.70	6.60	30.20	17.50	33.10	124.00	51.00	100.00	3.65	6.26	2.61	22.32	90.42	4.73
171	*R*_40-*L*3_	19.00	4.00	49.50	8.60	18.00	53.00	120.00	45.00	80.00	2.20	3.77	1.57	31.93	80.91	5.04
172	*R*_41-42_	35.50	6.20	8.50	31.60	7.70	58.20	327.00	97.00	156.00	0.90	1.54	0.64	29.75	194.03	5.30
173	*R*_41–43_	34.90	8.30	33.30	19.00	3.70	39.80	65.00	45.00	133.00	1.65	2.83	1.18	28.40	76.64	4.59
174	*R*_41-*L*2_	21.00	4.60	36.20	30.40	17.50	58.10	39.00	46.00	91.00	2.35	4.03	1.68	33.74	56.15	5.08
175	*R*_42-43_	18.40	3.90	31.60	11.60	4.70	41.80	90.00	28.00	57.00	0.80	1.37	0.57	23.78	57.68	4.10
176	*R*_42-*L*2_	21.60	7.30	18.50	9.80	15.10	35.00	123.00	30.00	68.00	2.70	4.63	1.93	21.71	73.19	4.41
177	*R*_43-*L*2_	18.80	6.40	10.40	17.00	14.30	55.50	92.00	54.00	49.00	3.45	5.91	2.46	25.47	65.90	4.67
178	*R*_43-*L*3_	11.00	6.10	15.40	14.40	11.00	35.40	102.00	86.00	64.00	4.20	7.20	3.00	19.06	85.44	4.60

**Table 2:** Sum of PLs and ranking of CLT locations.

Number	Location	∑*Q*_EUV_	∑*Q*_EFV_	*L*_*i*_	Rank
1	GLT_1_	294.86	297.47	2.61	4
2	GLT_2_	290.12	294.30	4.18	3
3	GLT_3_	287.34	298.98	11.64	1
4	GLT_4_	289.62	296.71	7.09	2

## Appendix

See Table 1.
